# Predicting Outcomes of Impingement-Based Mass Spectrometry Imaging

**DOI:** 10.1021/acs.analchem.6c03821

**Published:** 2026-09-16

**Authors:** John C. Sentmanat, Andrei G. Fedorov

**Affiliations:** † G. W. Woodruff School of Mechanical Engineering, 1372 Georgia Institute of Technology, Atlanta, Georgia 30332, United States; ‡ Parker H. Petit Institute for Bioengineering and Bioscience, 1372 Georgia Institute of Technology, Atlanta, Georgia 30332, United States

## Abstract

A physics-based scaling framework to predict the outcomes of impingement-based mass spectrometry imaging (MSI) techniques is presented to understand the underlying mechanisms and guide performance improvements. Particular emphasis is placed on analyte extraction, the fundamental process that defines MSI resolution and the softness of ionization. Two distinct regimes, thermodynamic (passive) and kinematic (active), are identified that follow different physical laws. Thermodynamic desorption arises under conditions approaching thermal equilibrium, where molecules are extracted through favorable energetic interactions with the solvent phase. In contrast, kinematic desorption occurs when high-energy particles or droplets impact the sample, supplying mechanical energy to overcome the cohesive forces that bind molecules at the surface. A dimensionless parameter is developed that compares the stress generated by an impacting particle to the cohesive forces binding molecules within a sample, providing a criterion for identifying transitions among extraction regimes. Through a scaling analysis of charged particle transport and impact dynamics, this parameter is expressed in terms of experimentally controllable inputs, revealing that kinematic extraction becomes possible only when the accelerating electric force exceeds the combined effects of the aerodynamic drag and particle inertia. Application of the methodology to representative MSI techniques yields predictions consistent with experimentally observed extraction behaviors, which supports the proposed mechanistic interpretation. More broadly, this framework establishes a semiquantitative foundation for predicting molecular extraction across a wide range of solution- and impingement-based MSI platforms, enabling systematic exploration of performance limits and guiding the design of next-generation MSI technologies using fundamental experimentally measured properties.

## Graphical abstract

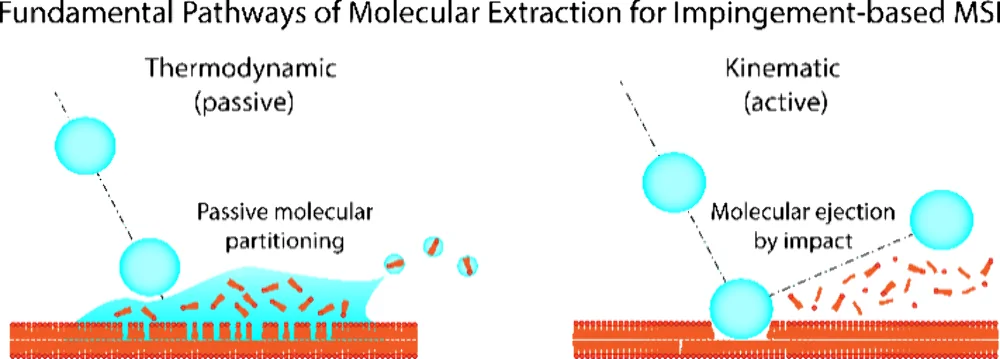

## Introduction

There has been tremendous growth in mass spectrometry imaging (MSI) over the past several decades, driven by its increasing ability to spatially resolve molecular distributions at the single-cell level^1,2^ while maintaining exceptional chemical specificity and sensitivity when compared with other imaging modalities. Matrix-assisted laser desorption/ionization (MALDI) has been the workhorse among MSI platforms, but solution-based (e.g., desorption electrospray ionization (DESI), nano-DESI, liquid microjunction-surface sampling probe (LMJ-SSP)) and particle impingement-based (e.g., secondary ion mass spectrometry (SIMS), gas cluster ion beam (GCIB), and electrospray droplet impact (EDI)) probing of cells and tissues have been growing over the past decade. Unlike MALDI, which uses laser energy to thermally desorb/ablate and ionize molecules, these techniques rely on interactions between particles, droplets, or solvent systems and complex biological surfaces to extract and ionize a broad range of analytes including lipids, metabolites, peptides, and intact proteins. Despite their diversity, all impingement-based MSI techniques must address a fundamental trade-off among spatial resolution, biomolecular coverage, and ionization softness. Consequently, there is a critical need for a physically motivated scaling framework, grounded in the fundamental principles of thermodynamics, fluid mechanics, and particle dynamics, that can relate controllable operating parameters to the molecular extraction behavior. This work presents a physics-based scaling framework for impingement-based MSI techniques that identifies the dominant analyte extraction mechanism and provides qualitative guidance regarding fragmentation propensity and the trade-off between spatial resolution and extraction softness.

Broadly, these techniques can be categorized into two distinct analyte extraction regimes: thermodynamic and kinematic. Thermodynamic (passive) desorption describes molecule removal for a system in thermal equilibrium.^3^ DESI is the prototypical thermodynamic desorption MSI technique and operates through a two-step extraction mechanism.^4^ Primary droplets impact the sample to form a liquid film (Figure 1A), allowing soluble surface molecules to partition into the liquid phase, driven by a favorable chemical potential gradient. Subsequent droplet impacts generate splashing events that eject analyte-laden charged secondary droplets from the liquid film on a sample surface for subsequent mass spectrometry detection.^5^ This ‘droplet-pickup’ removal mechanism minimizes molecular fragmentation, making DESI particularly suitable for the analysis of fragile biomolecules. However, the requirement for a favorable chemical potential gradient necessitates careful spray solvent selection to preferentially extract targeted analytes, which can limit the desorption efficiency of less soluble molecular species.^6^ Furthermore, the presence of a liquid film often constrains the achievable spatial resolution, as sufficient surface wetting is required for effective molecule extraction.^7^

**Figure 1:**
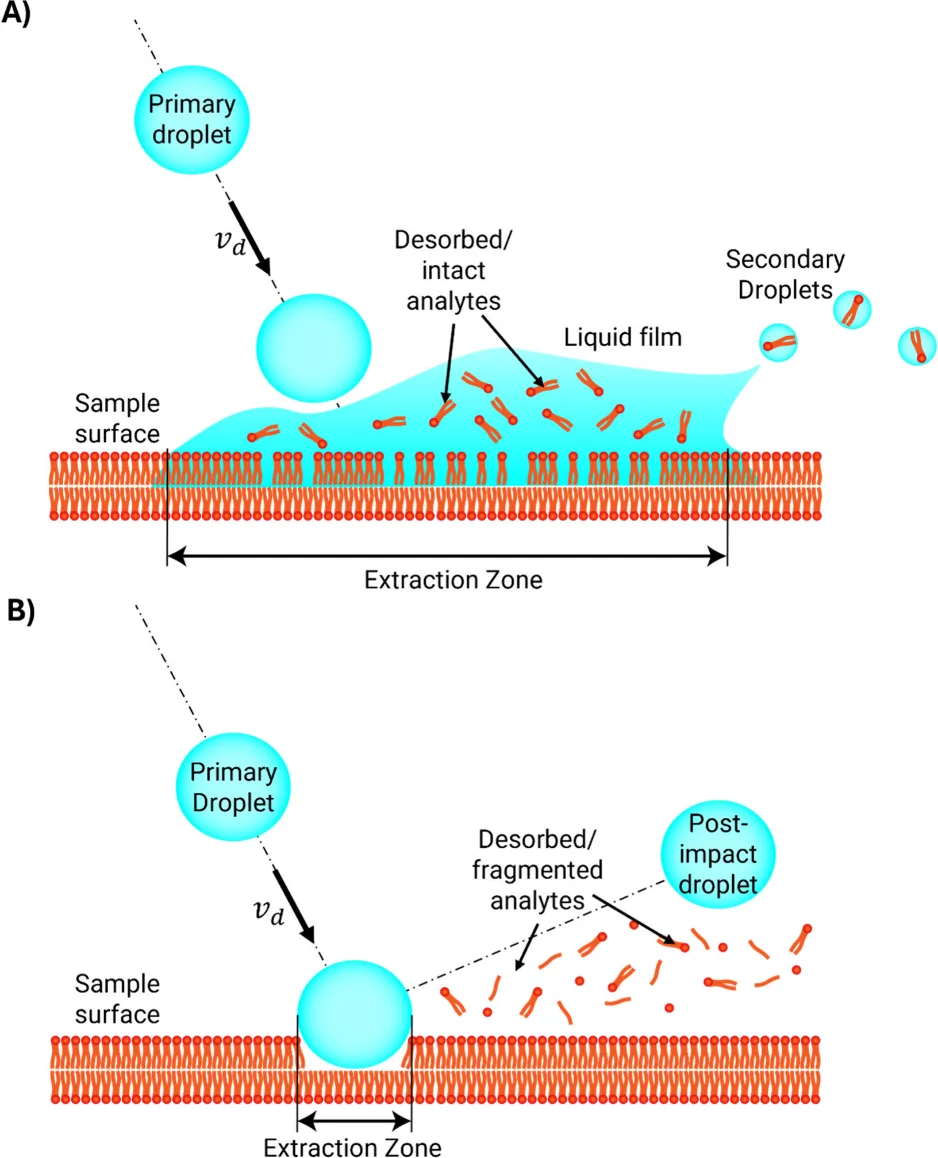
The key fundamental elements of two molecular extraction mechanisms considered in the proposed framework for impingement-based MSI. Analyte extraction from the sample surface requires overcoming the interactions that bind molecules within their surrounding environments. (A) Thermodynamic (passive) desorption occurs under thermal equilibrium and requires a favorable chemical potential gradient to allow molecules to freely desorb. (B) Kinematic (active) extraction uses high energy particles to break intermolecular interactions and eject molecules from the sample matrix.

In contrast, kinematic (active) extraction refers to impact-driven molecule removal by high-energy charged droplets or particles that mechanically separate and eject molecules from the sample. EDI, GCIB, and massive cluster impact (MCI) are representative kinematic desorption techniques that have been implemented for SIMS. In these techniques, large clusters or droplets impact the sample at supersonic velocities, ejecting surface molecules (Figure 1B). A shock wave induced within the sample upon impact has been hypothesized to break intermolecular interactions within the sample matrix, enabling molecules to be ejected.^8,9^ Owing to the rapid time scale of shock propagation, such impacts can partially preserve intact molecules as large as small proteins (<12 kDa).^10^ However, when impacting particles become sufficiently small, as in monatomic or small cluster SIMS ion sources, shock waves may no longer form due to environment rarefication. Under these conditions, the impact energy is transferred directly to the internal energy of the bound molecules, which may promote molecular fragmentation through bond cleavage.

Numerous experimental parameters influence the performance of the impingement-based MSI techniques, and transitions between thermodynamic and kinematic extraction regimes can substantially alter the types of biomolecules that are successfully extracted and analyzed as well as the degree of analyte fragmentation. The mechanistic framework that we present seeks to provide physically meaningful guidance to the design and optimization of MSI analyte extraction. Our methodology is based on employing fundamental conservation laws to establish a physics-based scaling framework that helps improve the performance of existing impingement-based imaging methods and assists in the development of next-generation platforms with minimum empiricism.

## Mechanistic Analysis and Scaling Framework

Analyte extraction behavior resulting from charged droplet/particle impacts can be predicted using scaling of momentum and energy conservation equations that relate the dynamics of the impacting particle to the cohesive forces binding molecules within their sample environment. This framework can be applied to a wide range of charged particle–surface interactions, and here, we demonstrate it for a common representative system consisting of an impinging water droplet extracting a lipid from a lipid monolayer.

Consider a charged droplet impacting the sample matrix with a given velocity (Figure 2). Kinematic extraction becomes possible when the stress generated during droplet impact exceeds the cohesive forces binding analytes within the sample. The impact stress, σ_*impact*_, corresponds to the dynamic pressure generated at impact whereas the cohesive stress, σ_*desorb*_, can be estimated from the energy required to remove a molecule from its bound state per its cross-sectional area for a given removal distance. This energy can be found empirically or computed by molecular dynamics (MD) simulations, as is the case for lipid desorption, which can be identified as the potential mean force (PMF) of a lipid moving from its equilibrium position within the bilayer to the free environment.^11,12^ Equating the impact stress and cohesive stress defines a critical impact velocity above which kinematic analyte extraction becomes possible. Below this critical velocity, analyte desorption is expected to occur primarily through thermodynamic mechanisms. Thus, a dimensionless parameter, Π_σ_, can be defined to predict the threshold for onset of kinematic extraction by comparing the magnitude of the impact and cohesive stresses, eq 1.

$$\Pi_{\sigma }=\frac{\rho_{p}v_{p}^{2}A_{m}l}{\Delta G_{desorb}}$$ 1

where ρ_*p*_ is the density of the particle, *v*_*p*_ is the particle velocity, *A*_*m*_ is the cross-sectional area of the molecule, *l* is the distance required to remove the molecule from its bound environment, and Δ*G*_*desorb*_ is the energy required to remove the molecule. For Π_σ_ < 1, extraction is only possible through thermodynamic desorption, and for Π_σ_ ≥ 1, kinematic desorption becomes possible. The threshold Π_σ_ ≥ 1 should not be interpreted as a sharp transition between these extraction mechanisms; rather, it marks the onset at which kinematic desorption can contribute to analyte extraction. As Π_σ_ increases beyond unity, kinematic desorption is expected to become increasingly dominant, while values near Π_σ_∼1 redefine a mixed-regime zone in which both thermodynamic and kinematic extraction mechanisms may coexist.

**Figure 2:**
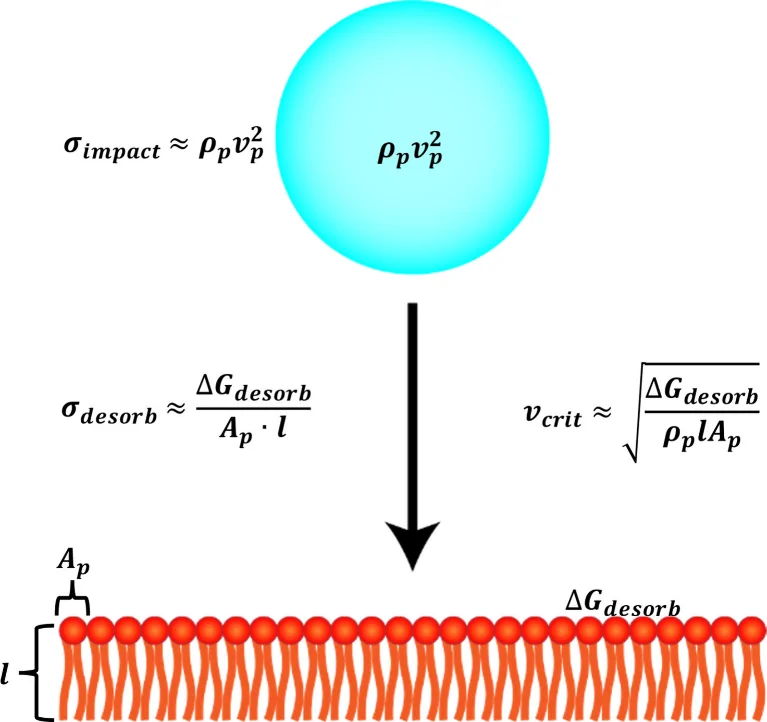
The physical basis of the proposed impact-stress framework for impingement-based molecular extraction. Kinematic extraction occurs when the stress generated during droplet or particle impact exceeds the cohesive forces binding analyte molecules within the sample matrix.

To illustrate how the dynamics of the impacting particle affects the analyte extraction outcome, the particle motion follows Newton’s second law, eq 2. The right-hand side of the equation contains the electric force due to the electric field accelerating the particle and the drag experienced by the particle from the surrounding environment. Here, *m*_*p*_ is the particle mass, $\overset{⃗}{v_{p}\;}$ is the particle velocity, *q* is the charge on the particle, E⃗ is the external electric field vector, *C*_*D*_ is the drag coefficient,^13^ ρ_*g*_ is the density of the surrounding gas, *A*_*p*_ is the particle cross-section area, and $\overset{⃗}{v_{g}\;}$ is the gas velocity vector.

$$m_{p}\frac{d\overset{⃗}{v_{p}\;}}{dt}=q\mathrm{E⃗}-\frac{1}{2}C_{D}\rho_{g}A_{p}\left(\overset{⃗}{v_{p}\;}-\overset{⃗}{v_{g}\;}\right)\left|\overset{⃗}{v_{p}\;}-\overset{⃗}{v_{g}\;}\right|$$ 2

For a 1D impact, the equation of motion can be nondimensionalized to identify scaling terms, eq 3.

$$\frac{dv^{*}}{dt^{*}}=\frac{qE_{s}L_{s}}{mv_{s}^{2}}E^{*}+\frac{C_{D}\rho_{g}A_{p}L_{s}}{2m_{p}}v^{*}\left|v^{*}\right|$$ 3

where *v*_*s*_, *E*_*s*_, and *t*_*s*_ = *L*_*s*_/*v*_*s*_ are identified as relevant scales for each parameter. The scales should be chosen to represent the threshold at which kinematic extraction becomes possible for impingement-based MSI, eq 4.

$$v_{s}=v_{crit},E_{s}=E_{pp},L_{s}=d_{sep}$$ 4

where *v*_*s*_ is taken as the critical velocity for kinematic desorption, and *L*_*s*_ is taken as the separation distance between the charged particle source and impact location. *E*_*s*_ can be approximated as a parallel plate electric field between the charged particle source and sample (counter electrode): *E*_*pp*_∼Δϕ/*d*_*sep*_, where Δϕ is the electric potential difference, and *d*_*sep*_ is the separation distance. For large distances between the charged particle source and the impact location, the parallel plate electric field approximation has been shown to be a good estimate of more complex electric field geometries.^14^ From these definitions, two dimensionless parameters can be defined that describe the relative magnitude of forces acting on the impacting particle by comparing the electric force and drag to particle inertia (pseudoforce) in eq 5a and 5b, respectively.

$$\Pi_{1}=\frac{q\Delta \phi }{mv_{crit}^{2}}$$ 5a

$$\Pi_{2}=\frac{C_{D}\rho_{g}A_{p}d_{sep}}{2m_{p}}$$ 5b

The relative magnitude of these dimensionless groups, Π_1_ and Π_2_, indicates which forces dominate impact dynamics and enables definition of the map for passive (thermodynamically driven) vs active (kinematically driven) regimes of analyte extraction.

## Discussion

Figure 3A shows the comprehensive map of Π_1_ vs Π_2_ for over 8 orders of magnitude variation, encompassing the range of conditions commonly encountered in impingement-based MSI. Because the governing extraction mechanism in MALDI relies on laser-mediated thermal desorption/ablation rather than particle or droplet impact, it falls outside the scope of the mechanical impact-stress framework developed in this study. Broadly, kinematic extraction becomes possible when the electric force acting on a particle exceeds the combined effects of drag and inertia (that is when Π_σ_ > 1). For Π_2_ < 1, where drag is insignificant relative to inertia, kinematic extraction occurs only when the electric force exceeds the particle inertia, corresponding to Π_1_ being of the order unity (Π_1_ = *O*(1)). EDI-based,^15–19^ GCIB-based,^20–23^ , and MCI-based^10,24,25^ SIMS ion sources fall within this regime because relatively small particles are accelerated through a low-drag vacuum environment prior to impact onto the sample surface. The locations of the different MSI techniques on the regime map correspond to representative operating conditions measured experimentally and reported in the literature (Table S1). Because these operating parameters can vary substantially between instruments and experimental conditions, the plotted locations should be interpreted as representative of the governing extraction regime and associated trends rather than as unique or fixed operating points for each technique.

**Figure 3:**
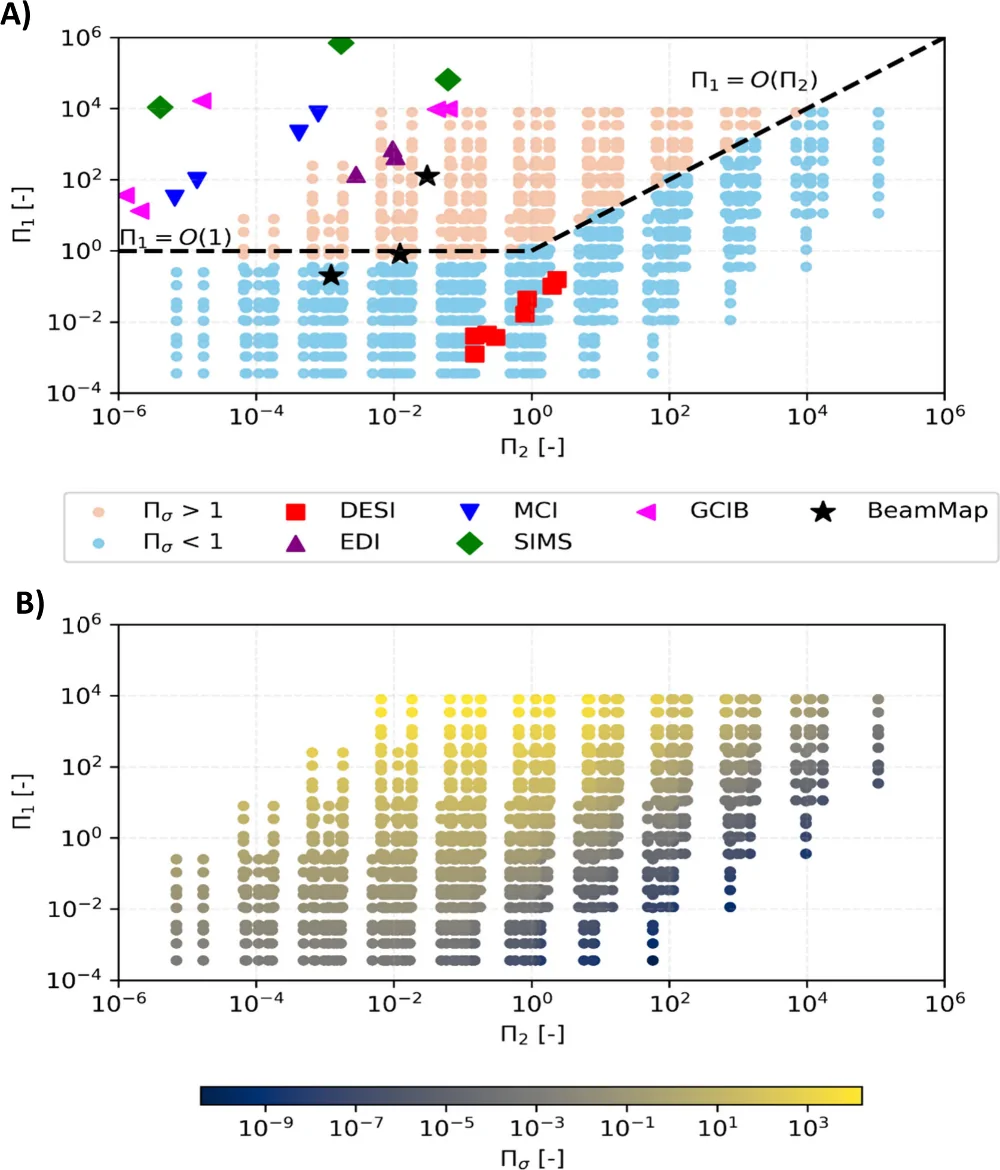
Regime map classifying impingement-based molecular extraction into thermodynamic and kinematic regimes based on the relative balance of electric, drag, and inertial forces. Kinematic extraction becomes possible when the electric force on a particle exceeds the combined effects of drag and inertia. (A) Plotting for several orders of magnitude of dimensionless parameters Π_1_ and Π_2_, kinematic and thermodynamic regimes are separated along the borderlines: Π_1_ = *O*(1) for Π_2_ < 1 and Π_1_ = *O*(Π_2_) for Π_2_ > 1. Using operating parameters from experiments, different MSI techniques are placed on the regime map (see markers), which indicates their primary analyte extraction mechanism (e.g., thermodynamic for DESI and kinematic for EDI). The ability to tune the relative magnitude of forces acting on the charged droplet characteristic to the BeamMap MSI technique allows operation in both the thermodynamic and kinematic regimes, opening the window for optimizing the trade-off between the imaging resolution and softness of analyte ionization. (B) The harshness of active (kinematically driven) analyte extraction increases as the electric force becomes increasingly dominant relative to drag and inertia, as graphically indicated by the color map of dimensionless Π_σ_ magnitude, defining the operating regimes which result in greater analyte fragmentation during extraction.

For Π_2_ > 1, when drag is significant relative to inertia, kinematic extraction becomes possible only when the electric force is greater than the drag force, corresponding to Π_1_ scaling with Π_2_ (Π_1_ = *O*(Π_2_)). In this drag dominant regime, the domain for thermodynamically driven desorption expands to progressively larger values of Π_1_ > 1 as Π_2_ increases. Physically, as drag exerts greater control over particle momentum, increasingly larger electric forces are required to achieve the impact conditions necessary for kinematic analyte extraction. DESI^4,26–32^ falls within this passive (thermodynamically driven) desorption regime because it operates at atmospheric pressure, where drag strongly influences the transport of comparatively large electrospray droplets typical of the technique.

Although other solution-based MSI approaches, such as nano-DESI and LMJ-SSP, cannot be directly represented on the regime map since they do not rely on impinging particles, they nevertheless operate through thermodynamically driven desorption and therefore are subject to the same limitations on the imaging resolution. The framework developed here is intended primarily for impingement-based MSI techniques; consequently, any extension to nano-DESI or LMJ-SSP should be interpreted as a qualitative approximation rather than a rigorous mechanistic description. For these systems, the regime transition parameter Π_σ_ can still be used and estimated by replacing the particle velocity, *v*_*p*_, in eq 1 with the average solution velocity, calculated from the volumetric flow rate per the liquid-surface contact area. Using this approach, Π_σ_ is on the order of ∼10^–9^ for nano-DESI^33^ and ∼10^–11^ for LMJ-SSP,^34^ both falling well within the thermodynamic desorption regime. While these estimates should not be interpreted quantitatively, they suggest that the underlying scaling concepts may provide qualitative insight into the operating behavior of related liquid extraction-based methods.

The magnitude of Π_σ_ increases as the electric force comes to dominate particle transport (Figure 3B). Monoatomic or small particle-based SIMS^35–37^ exhibits exceptionally large values of Π_σ_ because inertial forces are negligible due to the particle mass, while drag forces are effectively absent under vacuum conditions, allowing charged particles to accelerate unimpeded prior to impact. Consequently, these particles deliver a very high energy per impacting molecule, which leads to intramolecular fragmentation and challenges with analyzing labile compounds. Although Π_σ_ does not directly predict the threshold for molecular fragmentation, high values of this parameter are a predictive proxy of an increased likelihood of bond cleavage and fragmentation during extraction. We note, however, that ionization softness is governed not only by the extraction process but also by factors such as gas-phase ionization chemistry,^38,39^ molecular structure,^40^ internal energy redistribution,^41^ and local sample environment.^42^ Thus, a limitation of the present study is that these coupled processes are not experimentally resolved, and Π_σ_ should be interpreted as providing qualitative trends of fragmentation propensity rather than a quantitative predictor of ionization softness.

Future studies could aim for more directly distinguishing desorption from subsequent ionization and fragmentation by independently measuring material removal and mass spectrometric ion yield. For example, a fluorescent probe with well-characterized mass spectrometric behavior, such as rhodamine 6G,^17,30^ could be deposited on a surface and imaged by fluorescence microscopy before and after extraction to quantify spatially resolved material removal. The corresponding ion yield and fragmentation pattern could then be measured simultaneously with mass spectrometry. Correlating the amount of material removed with the detected intact and fragment ion signals would help distinguish the contributions of the desorption efficiency from subsequent ionization and dissociation processes. Such measurements could provide a more quantitative assessment of how extraction dynamics influence the observed mass spectrum but are beyond the scope of this work.

Unique within this regime map is BeamMap,^43^ a recently developed multimodal MSI technique that bridges the aspects of both the passive (DESI) and active (EDI, MCI, and SIMS) approaches through a tunable vacuum electrospray ionization (vESI) source. The design objective of BeamMap is to combine the soft ionization associated with thermodynamically driven extraction with the high spatial resolution characteristic of kinematically driven approaches. Current experimental MSI demonstrations are limited to a raster spacing of 500 μm and therefore do not yet demonstrate subcellular chemical imaging. However, characterization of the vESI source has demonstrated beam diameters below 500 nm and impinging liquid spots of a few micrometers, indicating the potential for substantially finer extraction under optimized operating conditions. Based on the currently available experimental parameters, BeamMap occupies the transition region near Π_σ_∼1 on the regime map, where both thermodynamic and kinematic extraction mechanisms are expected to contribute. This operating regime provides a design pathway for pursuing higher spatial resolution while maintaining the relatively soft extraction conditions desirable for the analysis of larger biomolecules, such as proteins.

## Conclusions

Addressing the trade-offs among spatial resolution, chemical selectivity, and ionization softness remains a fundamental challenge in the development of impingement-based MSI techniques. Understanding the fundamental physics underlying the biomolecule removal, whether governed by thermodynamic (passive) or kinematic (active) analyte extraction, is essential for predicting imaging outcomes and maximizing the molecular information captured in complex biological samples. We present a mechanistic framework that links experimentally controllable inputs to analyte extraction behavior with no empirical fitting, providing a predictive tool for understanding the current MSI capabilities and guiding the optimization and design of next-generation MSI platforms.

Central to this framework is the dimensionless regime transition parameter, Π_σ_ = ρ_*p*_ *v*_*p*_^2^ *A*_*m*_ *l*/Δ*G*_*desorb*_, that relates the stress generated by impinging particles to the cohesive forces binding molecules within the sample matrix. When Π_σ_ ≥ 1, active kinematic extraction becomes possible; otherwise, passive molecular desorption occurs through thermodynamically driven processes. By expressing this parameter in terms of experimentally controllable inputs using the scaling analysis of charged particle transport, we show that kinematic extraction is achieved only when the accelerating electric force exceeds the combined effects of aerodynamic drag and particle inertia. Application of this methodology to representative MSI techniques yields predictions that are consistent with their experimentally observed behaviors, providing validation of the proposed mechanistic analysis.

More broadly, the proposed scaling framework establishes a semiquantitative foundation for understanding and predicting the mode of analyte extraction across a wide range of solution- and impingement-based MSI platforms. By linking physical behavior to experimentally controllable parameters, it enables systematic exploration of the performance limits of existing imaging techniques while providing design principles for next-generation MSI methods with a minimal empiricism. As MSI continues to advance toward single-cell spatial resolution and comprehensive multiomic analyses, the scaling frameworks derived from chemo-mechanical analyses such as the one presented here will play an increasingly important role in guiding instrument design, operating conditions, and analytical performance.

## Supplementary Material



## Data Availability

The data that supports the findings can be made available upon request

## Notes

Table S1: Parameters used to construct the molecular extraction regime map, explanations of estimated values for missing information, and corresponding literature references to each data point collected (PDF)
