# Supplementary material for: Predicting Outcomes of Impingement-Based Mass Spectrometry Imaging
Source: Anal Chem. 2026 Sep 16;98(39):28617–22. doi: 10.1021/acs.analchem.6c03821 (PMC13647986; doi:10.1021/acs.analchem.6c03821)
Supplement: Supplementary file 1 [file ac6c03821_si_001.pdf]

Supporting Information

Predicting outcomes of impingement-based mass spectrometry imaging

John C. Sentmanat[1] and Andrei G. Fedorov[1,2*]

AFFILIATIONS

[1]G. W. Woodruff School of Mechanical Engineering, Georgia Institute of Technology, Atlanta, Georgia, 30332, USA

[2]Parker H. Petit Institute for Bioengineering and Bioscience, Georgia Institute of Technology, Atlanta, Georgia, 30332, USA

[*]Corresponding author: AGF@gatech.edu (Email); +1-404-385-1356 (Voice)

CONTENTS

Table S1. Parameters used to construct the molecular extraction regime map for representative impingement-based MSI techniques……………………………………………………………S-2

Technique	Particle/Solvent	Molecules analyzed	Velocity [m/s]	Particle radius [m]	Particle density [kg/m^3]	Voltage [V]	Separation distance [m]	Pressure [Pa]	Particle charge [C]	Particle mass [kg[	Particle energy [eV]	Πσ [-]	Π1 [-]	Π2 [-]	Ref.
DESI	1:1 MeOH:H2O	Melittin, Rhodamine B	140	2.00E-06	900	5000	2.00E-03	1.01E+05	1.40E-14	3.02E-14	1.84E+09	**1.32E-01**	**4.45E-02**	**8.70E-01**	Droplet Dynamics and Ionization Mechansms in Desorption Electrospray Ionization Mass Spectrometry
DESI	ACN	rhodamine-6G	166	1.26E-05	792	4000	2.00E-03	1.01E+05	3.53E-13	6.67E-12	5.73E+11	**1.64E-01**	**1.25E-03**	**1.50E-01**	Improved imaging resolution in desorption electrospray ionization mass spectrometry
DESI	ACN, 4:1 ACN:H2O w/0.2 Formic Acid	Rodent brain, kidney, ovarian, and breast tissue (Hemoglobin, S100A6, S100A6)	160	2.87E-06	786	5000	2.00E-03	1.01E+05	4.34E-14	7.80E-14	6.24E+09	**1.51E-01**	**1.64E-02**	**8.00E-01**	Desorption Electrospray Ionization Mass Spectrometry Imaging of Proteins Directly from Biological Tissue Sections
DESI	MeOH	rhodamine-6G	120	6.71E-06	792	5000	1.50E-03	1.01E+05	1.55E-13	1.00E-12	4.50E+10	**1.16E-01**	**4.60E-03**	**2.25E-01**	Deconstructing Desoprtion Electrospray Ionization: Independent Optimization of Desorption and Ionization by Spray Desoprtion Collection
DESI	1:1 MeOH:H2O	Fatty acids from E. coli, S. aureus, Enterococcus sp., B. thuringiensis, B. bronchiseptica, B. subtilis, S. typhimurium	1.80E+02	8.24E-06	925	6000	2.00E-03	1.01E+05	2.11E-13	2.17E-12	2.19E+11	**2.25E-01**	**4.05E-03**	**1.50E-01**	Desorption electrospray ionization mass spectrometry of intact bacteria
DESI	1:1 ACN:H2O w/0.1% Formic Acid	rhodamine-6G	1.00E+02	5.49E-07	910	4000	1.00E-03	1.01E+05	3.62E-15	6.29E-16	1.96E+07	**6.83E-02**	**1.57E-01**	**2.36E+00**	Detection of Attomole Amounts of Analyte by Desorption Electrospray Ionization Mass Spectrometry (DESI-MS) Determined Using Fluorescence Spectroscopy
DESI	MeOH	Rhodamine B	1.40E+02	6.71E-06	792	4000	2.00E-03	1.01E+05	1.55E-13	1.00E-12	6.12E+10	**1.16E-01**	**3.68E-03**	**3.01E-01**	Scanning and Surace Alignment Considerations in Chemical Imaging with Desorption Electrospray Mass Spectrometry
DESI	96:4 MeOH:H2O w/0.1% Formic Acid	PC	2.00E+01	4.32E-07	798	2000	5.00E-04	1.01E+05	2.30E-15	2.70E-16	3.37E+05	**2.39E-03**	**1.02E-01**	**1.98E+00**	Delineation of Subcellular Molecular Heterogeneity in Single Cells via Ultralow-Flow-Rate Desorption Electrospray Ionization Mass Spectrometry Imaging
EDI	1 M AcOH in H2O	Insulin, gramicidin S, arginine, cytochrome C, lysozyme	10000	8.60E-09	1000	10000	3.00E-01	1.33E-02	1.60E-17	2.66E-21	8.30E+05	**7.50E+02**	**4.50E+02**	**1.06E-02**	Fundamental aspects of electrospray droplet impact/SIMS
EDI	1 M AcOH in H2O	Au-S-(CH2)6-NH2, Rhodamine B, CsI, Crystal Violet	3000	8.63E-09	1000	10000	3.00E-01	1.33E-02	1.60E-17	2.69E-21	7.56E+04	**6.75E+01**	**4.46E+02**	**1.05E-02**	Energy dependence of projectiles on ion formation in electrospray droplet impact SIMS
EDI	1 M AcOH in H2O	Arginine, Lysine, Histidine	1.10E+04	5.00E-09	1005	10000	3.00E-01	1.33E-02	9.93E-18	9.96E-22	3.75E+05	**9.12E+02**	**7.51E+02**	**9.57E-03**	Study on ion formation in electrospray droplet impact secondary ion mass spectrometry
EDI	1 M AcOH in H2O	Arginine, Lysine, Histidine	1.10E+04	1.40E-08	1005	10000	3.00E-01	1.33E-02	4.98E-17	2.66E-20	1.00E+07	**9.12E+02**	**1.41E+02**	**2.81E-03**	Study on ion formation in electrospray droplet impact secondary ion mass spectrometry
EDI	H2O	HMTD, RDX, HMX, TNT, PETN	12000	8.63E-09	1000	10000	3.00E-01	1.33E-02	1.60E-17	2.69E-21	1.00E+06	**1.08E+03**	**4.44E+02**	**1.05E-02**	Electrospray droplet impact/secondary ion mass spectrometry (EDI/SIMS) applied to the analysis of explosives
GCIB	Ar60	Angiotensin III. Irganox 1010, PS1000	3.47E+04	8.41E-10	1600	20000	3.00E-01	6.67E-03	1.60E-19	3.98E-24	1.50E+04	**1.44E+04**	**9.60E+03**	**4.10E-02**	TOF-SIMS with Argon Gas Cluster Ion Beams: A Comparison with C60+
GCIB	C60	HeLa cells (membrane lipid fragments)	1.03E+05	5.57E-10	1650	6000	3.00E-01	6.67E-03	1.60E-19	1.2E-24	4.00E+04	**1.31E+05**	**9.90E+03**	**6.08E-02**	A New Dynamic in Mass Spectral Imaging of Single Biological Cells
GCIB	CO2	PE, PS, PI, LPI	5.01E+03	4.28E-09	1562	10000	3.00E-01	1.00E-06	1.60E-19	5.11E-22	4.00E+04	**2.94E+02**	**3.68E+01**	**1.24E-06**	Mapping the Molecular Landscape of Human DLBCL by GCIB-SIMS
GCIB	H2O	Glucose, PI	5.00E+03	5.98E-09	1000	10000	5.33E-01	1.00E-06	1.60E-19	8.97E-22	7.00E+04	**1.88E+02**	**1.33E+01**	**2.02E-06**	Successive High-Resolution (H2O)n-GCIB and C60-SIMS Imaging Integrates Multi-Omics in Different Cell Types in Breast Cancer Tissue
GCIB	C60	Antibodies tagged to targets	1.04E+05	5.57E-10	1650	10000	5.33E-01	1.00E-06	1.60E-19	1.2E-24	4.00E+04	**1.34E+05**	**1.65E+04**	**1.62E-05**	Successive High-Resolution (H2O)n-GCIB and C60-SIMS Imaging Integrates Multi-Omics in Different Cell Types in Breast Cancer Tissue

Technique	Particle/Solvent	Molecules analyzed	Velocity [m/s]	Particle radius [m]	Particle density [kg/m^3]	Voltage [V]	Separation distance [m]	Pressure [Pa]	Particle charge [C]	Particle mass [kg[	Particle energy [eV]	Πσ [-]	Π1 [-]	Π2 [-]	Ref.
MCI	0.75 M NH4OAc in Glycerol	Insulin, Cytochrome C, Kaplan peptide, avian cadherin, tetragastrin, pentagetide, Val-Pro	1.21E+03	3.07E-08	1261	15000	1.00E-01	1.00E-04	3.2E-17	1.53E-19	7.00E+05	**1.38E+01**	**3.03E+01**	**6.58E-06**	Large biomolecule desorption by massive cluster impact
MCI	1.5 M NH4OAc in Glycerol		4470	1.45E-08	1261	20000	1.00E-01	1.00E-04	8.02E-18	1.60E-20	1.00E+06	**1.89E+02**	**9.48E+01**	**1.38E-05**	
MCI	Au400	Glycine, phenylalanine, arginine, gramcidin S	3.58E+04	1.17E-09	19320	10000	2.00E+00	1.00E-04	6.41E-19	1.3E-22	5.20E+05	**1.86E+05**	**7.14E+03**	**7.95E-04**	Molecular Colocalization Using Massice Gold Cluster Secondary Ion Mass Spectrometry
MCI	Au2800	Glycine, phenylalanine, arginine, gramcidin S	1.91E+04	2.24E-09	19320	10000	2.00E+00	1.00E-04	1.28E-18	9.11E-22	1.04E+06	**5.29E+04**	**2.04E+03**	**4.16E-04**	Molecular Colocalization Using Massice Gold Cluster Secondary Ion Mass Spectrometry
SIMS	Au+	Dynorphin, Gramicidin S	9.90E+04	1.46E-10	19320	10000	7.00E-01	1.00E-04	1.60E-19	3.27E-25	1.00E+04	**1.42E+06**	**7.03E+05**	**1.71E-03**	The Aun cluster probe in secondary ion mass spectrometry: influence of the projectile size and energy on the desorption/ionization rate from biomolecular solids
SIMS	C60	DPPC, Gramicidin S	1.79E+05	5.57E-10	1650	40000	3.00E-01	6.67E-03	1.60E-19	1.2E-24	1.20E+05	**3.97E+05**	**6.60E+04**	**6.08E-02**	TOF-SIMS Analysis Using C60. Effect of Impact Energy on Yield and Damage
SIMS	Ga	PS Oligomers	2.72E+04	6.40E-10	5904	10000	3.00E-01	1.00E-06	1.60E-19	6.48E-24	1.50E+04	**3.28E+04**	**1.09E+04**	**4.01E-06**	Ion detection efficiency in SIMS: dependencies on energy, mass and composition for microchannel plates used in mass spectrometry
BeamMap	DI w/0.5% AcOH	Reserpine	2.32E+02	2.50E-07	1000	1000	1.00E-04	1.33E+02	1.76E-15	6.54E-17	1.10E+07	**4.04E-01**	**2.01E-01**	**1.22E-03**	BeamMap: Beam Enabled Accurate Mapping and Molecular Analyte Profiling for topography and biochemistry spatial correlation
BeamMap	DI w/0.5% AcOH	Reserpine	5.76E+03	1.00E-08	1000	5000	1.00E-04	1.33E+02	1.40E-17	4.19E-21	4.34E+05	**2.49E+02**	**1.26E+02**	**3.03E-02**	BeamMap: Beam Enabled Accurate Mapping and Molecular Analyte Profiling for topography and biochemistry spatial correlation
BeamMap	98:2 MeOH:DI w/0.5% Formic Acid	Reserpine	4.60E+02	2.50E-07	1000	4000	1.00E-03	1.33E+02	1.76E-15	6.54E-17	4.32E+07	**1.59E+00**	**8.05E-01**	**1.22E-02**	BeamMap: Beam Enabled Accurate Mapping and Molecular Analyte Profiling for topography and biochemistry spatial correlation

Table S1. Parameters used to construct the molecular extraction regime map for representative impingement-based MSI techniques. Literature-reported operating parameters were compiled to calculate the dimensionless scaling parameters Π_1, Π_2, and Π_σ for each technique. When parameters were not explicitly reported in the original references, values were estimated using the procedures described below. These estimates are intended to provide representative operating conditions in terms of dimensionless parameters for placement on the regime map and should not be interpreted as unique values for a given technique.

- Particle velocity: For DESI-based investigations, droplet velocity was estimated using the correlation between droplet velocity and nebulizing gas pressure reported by Venter *et al.* (2006)[1]. For other particle-based impingement methods, particle velocity was calculated from the reported particle kinetic energy and mass according to $v_p = \sqrt{2E_k/m_p}$, where E_k is the kinetic energy of the particle and m_p is the particle mass.
- Particle radius: For DESI and other droplet-based impingement methods, droplet radius was estimated using the electrospray scaling relationship of De La Mora and Loscertales (1994)[2], $R_d \approx \left(V_f \varepsilon/\kappa\right)^{1/3}$, where V_f is the volumetric flow rate, ε is the solution electrical permittivity, and κ is the solution electrical conductivity.
- Separation distance: When the particle acceleration distance was not specified, it was estimated from typical accelerating quadrupole or ion-gun geometries used in SIMS-based instrumentation.
- Pressure: For DESI investigations in which droplet charge was not reported, the charge was estimated as 70% of the Rayleigh charge limit[3]: $q_{Ry} = 8\pi\sqrt{\gamma\varepsilon_0 R_d^3}$, where γ is the solvent surface tension and ε_0 is the permittivity of vacuum.
- Particle energy: For DESI investigations in which droplet kinetic energy was not reported, it was calculated from the estimated droplet mass and velocity according to: $E_k = 1/2mv_d^2$.

(1) Venter, A.; Sojka, P. E.; Cooks, R. G. Droplet dynamics and ionization mechanisms in desorption electrospray ionization mass spectrometry. *Analytical Chemistry* **2006**, *78* (24), 8549-8555.
(2) De La Mora, J. F.; Loscertales, I. G. The current emitted by highly conducting Taylor cones. *Journal of fluid mechanics* **1994**, *260*, 155-184.
(3) Rayleigh, L. XX. On the equilibrium of liquid conducting masses charged with electricity. *The London, Edinburgh, and Dublin Philosophical Magazine and Journal of Science* **1882**, *14* (87), 184-186.
